# Impact of gestational age on risk of cerebral palsy: unravelling the role of neonatal morbidity

**DOI:** 10.1093/ije/dyab131

**Published:** 2021-06-28

**Authors:** Ruoqing Chen, Arvid Sjölander, Stefan Johansson, Donghao Lu, Neda Razaz, Kristina Tedroff, Eduardo Villamor, Sven Cnattingius

**Affiliations:** 1 Clinical Epidemiology Division, Department of Medicine Solna, Karolinska Institutet, Stockholm, Sweden; 2 Department of Medical Epidemiology and Biostatistics, Karolinska Institutet, Stockholm, Sweden; 3 Department of Clinical Science and Education, Södersjukhuset, Stockholm, Sweden; 4 Department of Epidemiology, Harvard T.H. Chan School of Public Health, Boston, MA, USA; 5 Department of Women's and Children's Health, Karolinska Institutet, Stockholm, Sweden; 6 Department of Epidemiology, School of Public Health, University of Michigan, Ann Arbor, MI, USA

**Keywords:** Preterm birth, neonatal morbidity, cerebral palsy, mediation analysis

## Abstract

**Background:**

The contribution of adverse consequences of preterm birth to gestational-age-related risk of cerebral palsy (CP) has rarely been studied. We aimed to assess the potential mediating roles of neonatal morbidity on the association between gestational age and risk of CP.

**Methods:**

In this Swedish population-based study, 1 402 240 singletons born at 22–40 gestational weeks during 1998–2016 were followed from day 28 after birth for a CP diagnosis until 2017. Potential mediators included asphyxia, respiratory-related, infection-/inflammatory-related and neurological-related diseases within 0–27 days of life. Cox regression was used to estimate hazard ratios (HRs) and 95% confidence intervals (CIs). Causal mediation analysis was performed to estimate the proportion of the association mediated through pathways involving the four sequential mediators.

**Results:**

We found an inverse dose–response relationship between gestational age and risk of CP, where the strongest association was observed for 22–24 weeks (HR 47.26, 95% CI 34.09–65.53) vs 39–40 weeks. Compared with non-diseased peers, children with neonatal morbidity, particularly those with neurological-related diseases (HR 31.34, 95% CI 26.39–37.21), had a higher risk of CP. The increased risk of CP was, at 24 weeks, almost entirely explained by neonatal morbidity (91.7%); this proportion decreased to 46.1% and 16.4% at 32 and 36 weeks, respectively. Asphyxia was the main mediating pathway from 22 to 34 weeks, and neurological-related neonatal diseases led the mediating pathways from 34 weeks onwards.

**Conclusion:**

Neonatal morbidity mediates a large proportion of the effect of preterm birth on CP, but the magnitude declines as gestational age increases.

Key Messages

- Decreasing gestational age is associated with increasing risk of any cerebral palsy (CP) as well as different subtypes of CP, notably spastic diplegic CP.
- Neonatal morbidity, including asphyxia, respiratory-related diseases, infection-/inflammatory-related diseases and neurological-related diseases, is responsible for a large proportion of the increased risk of CP associated with decreasing gestational age, but the magnitudes decline as gestational age increases.
- Pathways involving asphyxia are the main mediating pathways from 22 to 34 weeks, whereas the pathway involving only neurological-related neonatal diseases plays the leading mediating role from 34 weeks onwards.
- Our findings suggest that prevention of neonatal morbidity may reduce the risk of CP and that the benefit of such a prevention increases with decreasing gestational age.

## Introduction

Cerebral palsy (CP) refers to a group of permanent and non-progressive disorders of movement and posture that results from insults to the brain.^1^ As the most common childhood-onset motor disability, CP affects between 1.5 and >3 per 1000 live births in the world.^1^ In western Sweden, the prevalence of CP decreased from 2.5 (1983–1986) to 2.0 (2007–2010) per 1000 live births.^2^^,^^3^ However, the global prevalence is relatively stable.^1^

Children born preterm are at an increased risk of CP.^4^ Due to advances in obstetric and neonatal care, the survival of preterm infants has increased in recent decades, which might also potentially explain the absence of a global decline in the CP prevalence.^1^ Therefore, the impact of adverse health consequences of preterm birth on the risk of CP needs to be thoroughly understood.

Neonatal asphyxia is recognized as one of the important CP risk factors.^5^ Other neonatal factors influencing the risk of CP include respiratory disorders, which may induce hypoxia, alteration in blood gas levels when using mechanical ventilation and hypocapnia,^6^ as well as central nervous system and systemic infections.^7^ Brain injuries and abnormal neurological events during the neonatal period are also associated with higher risk of CP.^1^^,^^5^ As preterm infants are exposed to the extrauterine environment too early, their immature organs are more susceptible to developing neonatal diseases.^8^ In spite of extensive research on the aetiology of CP, studies attempting to explore the mechanism of preterm birth leading to CP have been limited and never been performed in a causal inference framework.

This study was aimed to investigate the potential mediating role of neonatal morbidity, including asphyxia, respiratory-related diseases, infection-/inflammatory-related diseases and neurological-related diseases, on the association between gestational age and risk of CP in singleton children.

## Methods

### Study design and participants

We performed a population-based study of live-born singleton infants using data from the Swedish national registries, including Medical Birth, Patient, Cause of Death, Education, Total Population and Multi-Generation Registers. Details of data obtained from these registries are described in Supplementary Methods 1, available as Supplementary data at *IJE* online.

We identified 1 918 023 live singleton infants born during 1998–2016 from the Medical Birth Register. We excluded births with missing information on personal identity numbers of infants or mothers (*n* = 31 008), gestational age (*n* = 1059), sex (*n* = 2) and maternal age (*n* = 1). Infants delivered at 39–40 (i.e. 39 + 0 to 40 + 6) weeks of gestation have the lowest rates of adverse neonatal outcomes and CP.^9^^,^^10^ To study the potential dose–response relationship between decreasing gestational age and risk of CP, we excluded 481 077 infants born at ≥41 completed (i.e. 41 + 0) weeks. To study the possible mediating role of neonatal morbidity (i.e. diseases during the first 27 days of life), we further excluded infants who died (*n* = 2540) or emigrated (*n* = 93) during the first 27 days and infants who had a diagnosis of CP during the first 27 days but not again thereafter (*n* = 3). Our final study population included 1 402 240 children.

This study was approved by the Regional Ethical Review Board in Stockholm, Sweden (No. 2018/20–31/5).

### Exposure

Gestational age was estimated using the following hierarchy: ultrasonography offered no later than the early second trimester (89.8%); date of last menstrual period reported at the first antenatal visit (5.5%); and a postnatal assessment (4.8%). Gestational age was analysed as a continuous and a categorical variable. The categorization was first based on the definitions of extremely (22–27 weeks), very (28–31 weeks) and moderately (32–36 weeks) preterm births, and early (37–38 weeks) and full-term (39–40 weeks) births.^11^^,^^12^ We further split the extremely preterm births into 22–24 and 25–27 weeks, as infants born at 22–24 weeks have the highest neonatal morbidity and mortality.^13^^,^^14^ We also divided the moderately preterm births into 32–34 and 35–36 weeks, because infants born at 32–34 weeks are generally admitted to neonatal intensive care units, whereas those born at 35–36 weeks commonly stay in the maternity wards in Sweden.

### Confounders

Maternal characteristics included age at childbirth, parity, educational level, country of birth, smoking during pregnancy, height and weight. Body mass index (BMI) in early pregnancy was calculated as measured weight (kg) at the first antenatal visit divided by self-reported height squared (m^2^). Pregnancy complications included diabetic and hypertensive diseases, chorioamnionitis, infectious diseases and placental abruption [Supplementary Table S1, available as Supplementary data at *IJE* online, for the International Classification of Diseases, tenth revision (ICD-10) codes]. Mode of delivery was recorded in the obstetric records. Newborn characteristics included sex, major malformation, birthweight-for-gestational age and calendar year of birth. Major malformations were defined by diagnoses during the first year of age as recorded in the Medical Birth Register or Patient Register (Supplementary Table S1, available as Supplementary data at *IJE* online, for ICD-10 codes). We calculated z-scores of birthweight-for-gestational age based on the ultrasound-based, sex-specific Swedish reference curve for fetal growth.^15^ The z-scores were further converted to birthweight-for-gestational age percentiles.

### Mediators

**Figure 1:**
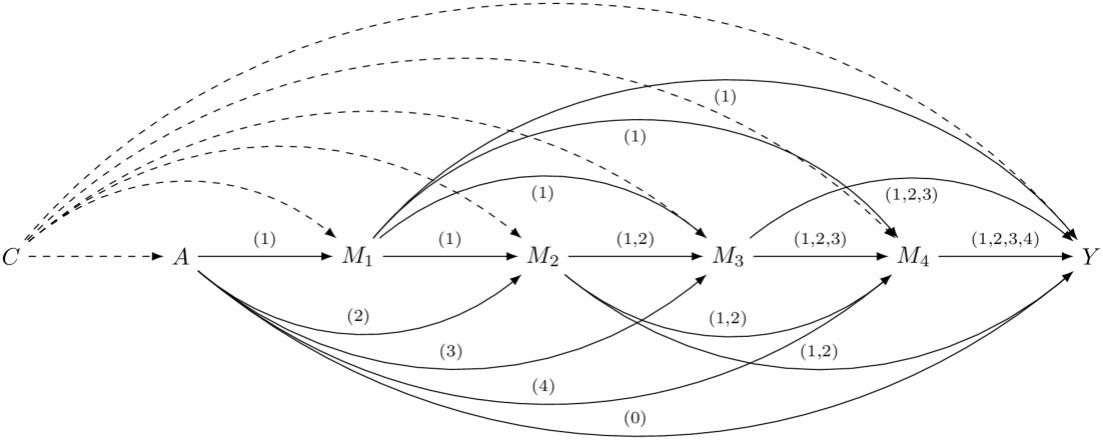
Directed acyclic graph to illustrate the possible structural relationship between gestational age, mediators, cerebral palsy (CP) and confounders. The arrow (0) represents the direct effect of A on Y, i.e. the effect that is not mediated through any of M1, M2, M3 or M4. All arrows (1)–(4) represent the natural indirect effect mediated through all pathways involving M1, M2, M3 and M4 jointly. All arrows (1) represent the natural indirect effect mediated through all pathways involving M1. All arrows (2) represent the partial indirect effect mediated through all pathways involving M2, but not through M1. All arrows (3) represent the partial indirect effect mediated through all pathways involving M3, but not through M1 or M2. The arrow (4) represent the partial indirect effect mediated through the pathway involving M4, but not through M1, M2 or M3. The dashed arrows represent the effect of C on A, M1, M2, M3, M4 and Y. A =gestational age; Y=CP; M1=asphyxia; M2=respiratory-related diseases; M3=infection-/inflammatory-related diseases; M4=neurological-related diseases; C=baseline confounders.

We considered neonatal morbidity (diagnosed at 0–27 days of age) related to gestational age as a potential mediator.^1^^,^^6^^,^^7^^,^^16–20^ We included asphyxia (intrapartum hypoxia and birth asphyxia) as Mediator 1 (M1); respiratory-related diseases (respiratory distress syndrome and bronchopulmonary dysplasia) as Mediator 2 (M2); infection-/inflammatory-related diseases (sepsis, other infections specific to the perinatal period, pneumonia, central nervous system inflammatory diseases and necrotizing enterocolitis) as Mediator 3 (M3); and neurological-related diseases (convulsion, intracranial non-traumatic haemorrhage and periventricular leukomalacia) as Mediator 4 (M4) (Supplementary Table S1, available as Supplementary data at *IJE* online, for ICD-10 codes). Development of respiratory-related neonatal diseases has been observed to be influenced by asphyxia^21^ and infants with asphyxia and respiratory-related diseases are more likely to develop neonatal infections.^22^^,^^23^ Asphyxia, respiratory-related and infection-related diseases can lead to injury and dysfunction of the brain.^24^^,^^25^ Therefore, we assumed an underlying causal structure where M2 is potentially influenced by M1, M3 is potentially influenced by M1 and M2, and M4 is potentially influenced by M1, M2 and M3 (Figure 1).

### Outcomes

CP was identified by a first clinical diagnosis of ICD-10 code G80 made in either hospitalization or outpatient visit during follow-up. We also considered risk of different CP subtypes associated with gestational age (Supplementary Table S1, available as Supplementary data at *IJE* online, for ICD-10 codes).

### Statistical analysis

Children were followed from day 28 after birth until the date of first diagnosis of CP, date of death, emigration, their 16th birthday or 31 December 2017, whichever came first. As our study included right censored time-to-event data, we used Cox regression with age as the underlying timescale. To assess the association between gestational age and CP, we estimated hazard ratios (HRs) with 95% confidence intervals (CIs). In the first adjusted model, maternal characteristics were included. The second adjusted model further incorporated pregnancy and newborn characteristics. Associations between gestational age and CP subtypes were also examined. To account for the correlation among siblings, we used a robust sandwich estimator of variance in all models.

To examine whether the studied association could be mediated by neonatal morbidity, we performed mediation analyses using the methods described by Steen *et al*.^26^ A detailed explanation of the mediation analyses is provided in Supplementary Methods 2, available as Supplementary data at *IJE* online. The directed acyclic graph in Figure 1 shows the relationships between the exposure, the four mediators, the outcome and the potential confounders. We estimated the following causal effects: (i) the total effect of gestational age on CP; (ii) the direct effect of gestational age on CP; (iii) the natural indirect effect mediated through all pathways involving M1, M2, M3 and M4 jointly; (iv) the natural indirect effect mediated through all pathways involving M1; (v) the partial indirect effect mediated through all pathways involving M2, but not through M1; (vi) the partial indirect effect mediated through all pathways involving M3, but not through M1 or M2, and (vii) the partial indirect effect mediated through the pathway involving M4, but not through M1, M2 or M3.

For the indirect effect components [(iii) to (vii)], we estimated the proportion of the total effect mediated by M1, M2, M3 and M4 [(iii) divided by (i)], by M1 [(iv) divided by (i)], by M2 but not M1 [(v) divided by (i)], by M3 but not M1 or M2 [(vi) divided by (i)] and by M4 but not M1, M2 or M3 [(vii) divided by (i)]. We assumed that all confounders listed above were sufficient to adjust for confounding for the associations between the exposure, the mediators and the outcome. We comment on the plausibility of this assumption in the ‘Discussion’ section.

All causal effects were defined as risk differences on the form *P(X)*–*P(40)*, where *P(X)* is the risk of developing CP before 5 years of age at a certain gestational age *X* and *P(40)* is the corresponding risk at 40 weeks of gestation (reference). Using 5 years of age was to achieve adequate evaluation of the risk of CP.

To estimate the causal effects, we fitted logistic-regression models for: (i) M1 as a function of gestational age and the confounders; (ii) M2 as a function of gestational age, M1 and the confounders; (iii) M3 as a function of gestational age, M1, M2 and the confounders; and (iv) M4 as a function of gestational age, M1, M2, M3 and the confounders. We fitted a Cox-regression model for time-to-CP as a function of gestational age, M1, M2, M3, M4 and the confounders. We used 100 bootstrap resamples to estimate 95% CIs.

Data preparation was performed using SAS version 9.4. Statistical analyses were performed using Stata version 15.1 and R version 3.5.2 (for mediation analyses).

## Results

A total of 3245 children were diagnosed with CP during a median follow-up of 9.4 years. The median age at diagnosis was 2.0 years (interquartile range: 1.1–3.8 years; maximum: 15.9 years). Risk of CP was increased among children of mothers with low and high age at childbirth, lower educational level, who smoked, had a short stature or had BMI <18.5 or ≥25.0 (Supplementary Table S2, available as Supplementary data at *IJE* online). Maternal diseases during pregnancy, emergency instrumental delivery, elective or unspecified caesarean section, male sex, newborn major malformations and low birthweight-for-gestational age (<10th percentile) were generally associated with higher risk of CP (Table 1). The distributions of maternal, pregnancy and newborn characteristics across gestational age are shown in Supplementary Table S3, available as Supplementary data at *IJE* online.

**Table 1:** Pregnancy and newborn characteristics and risk of cerebral palsy in children born at 22–40 weeks in Sweden during 1998–2016

Characteristics	No. of children (%)	Person-years	Cerebral palsy		
			No. of cases	Rate^a^	Hazard ratio (95% CI)^b^
Total	1 402 240	13 074 532	3245	2.48	
**Pregnancy**					
Diabetic diseases					
No	1 374 236 (98.00)	12 832 791	3125	2.44	Ref.
Pre-gestational diabetes	10 902 (0.78)	94 977	66	6.95	2.74 (2.14–3.50)
Gestational diabetes	17 102 (1.22)	146 763	54	3.68	1.46 (1.12–1.92)
Hypertensive diseases					
No	1 346 935 (96.06)	12 560 264	2990	2.38	Ref.
Pre-gestational hypertension	10 925 (0.78)	95 335	31	3.25	1.32 (0.93–1.88)
Pre-eclampsia	44 380 (3.16)	418 932	224	5.35	2.25 (1.96–2.58)
Chorioamnionitis					
No	1 399 109 (99.78)	13 048 316	3172	2.43	Ref.
Yes	3131 (0.22)	26 216	73	27.85	11.20 (8.88–14.12)
Infectious diseases during pregnancy					
No	1 359 925 (96.98)	12 775 792	3106	2.43	Ref.
Yes	42 315 (3.02)	298 740	139	4.65	1.62 (1.36–1.92)
Placental abruption					
No	1 396 394 (99.58)	13 018 929	3110	2.39	Ref.
Yes	5846 (0.42)	55 602	135	24.28	10.50 (8.82–12.51)
Mode of delivery					
Non-instrumental vaginal	1 075 533 (76.70)	10 067 972	1759	1.75	Ref.
Emergency instrumental^c^	177 135 (12.63)	1 658 194	784	4.73	2.71 (2.49–2.95)
Elective caesarean section	143 688 (10.25)	1 275 176	652	5.11	2.84 (2.60–3.11)
Unspecified caesarean section	5884 (0.42)	73 190	50	6.83	4.72 (3.56–6.26)
**Newborn**					
Sex					
Male	706 001 (50.35)	6 587 071	1857	2.82	1.31 (1.22–1.41)
Female	696 239 (49.65)	6 487 461	1388	2.14	Ref.
Major malformation					
No	1 339 949 (95.56)	12 539 930	2485	1.98	Ref.
Yes	62 291 (4.44)	534 601	760	14.22	6.87 (6.33–7.46)
Birthweight-for-gestational age (percentile)					
<3rd	35 606 (2.54)	322 981	374	11.58	5.54 (4.96–6.19)
3rd to <10th	83 416 (5.95)	756 575	311	4.11	1.94 (1.72–2.19)
10th to <90th	1 134 024 (80.87)	10 554 477	2208	2.09	Ref.
90th to <97th	89 638 (6.39)	862 867	151	1.75	0.85 (0.72–1.00)
≥97th	55 508 (3.96)	533 034	137	2.57	1.26 (1.06–1.49)
Missing	4048 (0.29)	44 597	64	14.35	
Calendar year of birth					
1998–2002	312 907 (22.31)	4 819 528	917	1.90	1.06 (0.96-1.16)
2003–2007	363 493 (25.92)	4 396 285	994	2.26	1.04 (0.95-1.14)
2008–2012	399 398 (28.48)	2 911 245	923	3.17	Ref.
2013–2016	326 442 (23.28)	947 474	411	4.34	0.82 (0.73-0.93)

a Rate is calculated as number of cases per 10 000 person-years.

b Unadjusted model.

c Instrumental vaginal delivery or emergency caesarean section.

With 39–40 weeks as the reference, the adjusted HR of CP consistently increased with decreasing gestational age in all models (Table 2). Although still markedly increased compared with the reference, gestational-age-specific HRs were attenuated with additional adjustment for pregnancy and newborn characteristics (Table 2, Model 3), primarily driven by adjustment for mode of delivery and major malformation (Supplementary Table S4, available as Supplementary data at *IJE* online). Spastic diplegic CP demonstrated the highest risk associated with decreasing gestational age (Supplementary Table S5, available as Supplementary data at *IJE* online).

**Table 2:** Gestational age and risk of cerebral palsy in children born at 22–40 weeks in Sweden during 1998–2016

Gestational age (weeks)	No. of children (%)	Person-years	Cerebral palsy				
			No. of cases	Rate^a^	Hazard ratio (95% CI)		
					Model 1^b^	Model 2^c^	Model 3^d^
As continuous variable (per 1-week decrease)	1 402 240 (100.00)	13 074 532	3245	2.48	1.36 (1.36–1.37)	1.36 (1.35–1.37)	1.29 (1.28–1.31)
As categorical variable							
22–24	832 (0.06)	5665	83	146.53	84.35 (67.35–105.63)	77.23 (58.01–102.81)	47.26 (34.09–65.53)
25–27	2744 (0.20)	23 169	197	85.03	53.96 (46.40–62.76)	52.00 (43.74–61.81)	24.18 (19.51–29.97)
28–31	8063 (0.58)	73 921	362	48.97	32.55 (28.97–36.58)	30.81 (27.05–35.09)	16.29 (13.69–19.39)
32–34	21 975 (1.57)	207 318	281	13.55	9.00 (7.91–10.24)	8.14 (7.04–9.40)	5.53 (4.72–6.49)
35–36	56 011 (3.99)	534 144	236	4.42	2.94 (2.56–3.38)	2.76 (2.37–3.21)	2.20 (1.88–2.57)
37–38	346 859 (24.74)	3 269 507	712	2.18	1.44 (1.31–1.57)	1.43 (1.30–1.57)	1.30 (1.18–1.44)
39–40	965 756 (68.87)	8 960 808	1374	1.53	Ref.	Ref.	Ref.

a Rate is calculated as number of cases per 10 000 person-years.

b Unadjusted model.

c Model 2 adjusted for maternal age at childbirth, parity, educational level, country of birth, smoking during pregnancy, height and early-pregnancy BMI.

d Model 3 adjusted for maternal age at childbirth, parity, educational level, country of birth, smoking during pregnancy, height, early-pregnancy BMI, diabetic diseases, hypertensive diseases, chorioamnionitis, infectious diseases during pregnancy, placental abruption, mode of delivery and child's sex, major malformation, birthweight-for-gestational age and calendar year of birth.

The prevalence of neonatal diseases increased with decreasing gestational age (Supplementary Table S6, available as Supplementary data at *IJE* online). Children with any neurological-related disease had a substantially higher risk of CP, followed by children with asphyxia, whereas those with any respiratory-related or infection-/inflammatory-related disease had more modestly increased risks of CP (Table 3).

**Table 3:** Neonatal morbidity (potential mediators) and risk of cerebral palsy in children born at 22–40 weeks in Sweden during 1998–2016

Neonatal morbidity (mediators)	No. of children (%)	Person-years	Cerebral palsy		
			No. of cases	Rate ^a^	Hazard ratio (95% CI)^b^
**Asphyxia**					
Intrapartum hypoxia					
No	1 401 574 (99.95)	13 066 610	3214	2.46	Ref.
Yes	666 (0.05)	7921	31	39.14	7.52 (4.69–12.04)
Birth asphyxia					
No	1 389 475 (99.09)	12 950 106	2733	2.11	Ref.
Yes	12 765 (0.91)	124 426	512	41.15	6.35 (5.51–7.31)
Any asphyxia					
No	1 389 091 (99.06)	12 945 467	2726	2.11	Ref.
Yes	13 149 (0.94)	129 065	519	40.21	6.31 (5.48–7.26)
**Respiratory-related diseases**					
Respiratory distress syndrome					
No	1 392 715 (99.32)	12 994 707	2780	2.14	Ref.
Yes	9525 (0.68)	79 825	465	58.25	1.50 (1.25–1.80)
Bronchopulmonary dysplasia					
No	1 399 969 (99.84)	13 057 904	3070	2.35	Ref.
Yes	2271 (0.16)	16 627	175	105.25	1.27 (1.01–1.61)
Any respiratory-related disease					
No	1 392 256 (99.29)	12 990 715	2750	2.12	Ref.
Yes	984 (0.71)	83 817	495	59.06	1.51 (1.26–1.82)
**Infection-/inflammatory-related diseases**					
Sepsis					
No	1 398 564 (99.74)	13 041 107	3072	2.36	Ref.
Yes	3676 (0.26)	33 425	173	51.76	1.56 (1.23–1.99)
Other infections specific to the perinatal period					
No	1 394 242 (99.43)	12 990 321	3148	2.42	Ref.
Yes	998 (0.57)	84 210	97	11.52	1.56 (1.23–1.97)
Pneumonia					
No	1 397 915 (99.69)	13 030 422	3193	2.45	Ref.
Yes	4325 (0.31)	44 109	52	11.79	1.90 (1.38–2.63)
Central nervous system inflammatory diseases					
No	1 401 955 (99.98)	13 072 468	3208	2.45	Ref.
Yes	285 (0.02)	2064	37	179.27	15.89 (8.76-28.81)
Necrotizing enterocolitis					
No	1 401 674 (99.96)	13 070 847	3194	2.44	Ref.
Yes	566 (0.04)	3685	51	138.41	2.06 (1.43–2.96)
Any infection-/inflammatory-related diseases					
No	1 386 199 (98.86)	12 913 575	2895	2.24	Ref.
Yes	16 041 (1.14)	160 956	350	21.75	1.96 (1.65–2.33)
**Neurological-related diseases**					
Convulsion					
No	1 399 780 (99.82)	13 055 227	2806	2.15	Ref.
Yes	2460 (0.18)	19 304	439	227.41	47.83 (41.17–55.57)
Intracranial non-traumatic haemorrhage					
No	1 399 955 (99.84)	13 058 123	2854	2.19	Ref.
Yes	2285 (0.16)	16 409	391	238.29	9.95 (8.03–12.32)
Periventricular leukomalacia					
No	1 402 043 (99.99)	13 073 525	3163	2.42	Ref.
Yes	197 (0.01)	1007	82	814.68	16.28 (11.27–23.51)
Any neurological-related diseases					
No	1 397 808 (99.68)	13 040 864	2485	1.91	Ref.
Yes	432 (0.32)	33 667	760	225.74	31.34 (26.39–37.21)
Any of the above morbidity					
No	1 365 622 (97.39)	12 720 835	2002	1.57	Ref.
Yes	36 618 (2.61)	353 696	1243	35.14	8.94 (7.86–10.17)

a Rate is calculated as number of cases per 10 000 person-years.

b Model adjusted for maternal age at childbirth, parity, educational level, country of birth, smoking during pregnancy, height, early-pregnancy BMI, diabetic diseases, hypertensive diseases, chorioamnionitis, infectious diseases during pregnancy, placental abruption, mode of delivery and child's sex, major malformation, birthweight-for-gestational age, calendar year of birth and gestational age (as categorical variable).

The results of mediation analyses are shown in Figures 2–1 and 2–2. Similar to results reported on the HR scale (Supplementary Table S7, available as Supplementary data at *IJE* online), the total effect of gestational age on CP before 5 years of age, reported on the risk-difference scale, increased as gestational age decreased (Figure 2–1A). The direct effect of gestational age on CP decreased with increasing gestational age and was small across all weeks (Figure 2–1B). The joint natural indirect effect mediated through the four mediators (Figure 2–1C) was similar to the total effect. For example, all pathways involving four mediators together potentially explained 91.7% of the increased risk of CP at 24 weeks compared with 40 weeks; this proportion decreased to 46.1% at 32 weeks and 16.4% at 36 weeks (Figure 2–1D). In lower gestational weeks, pathways involving asphyxia had a greater contribution than pathways involving respiratory-related diseases, infection-/inflammatory-related diseases and neurological-related diseases, respectively, the latter three having similar contributions to those of the mediators. From 34 weeks onwards, the pathway involving only neurological-related diseases led the mediating pathways, explaining most of the total effect of gestational age on CP among all mediators (Figure 2–2).

**Figure 2–1:**
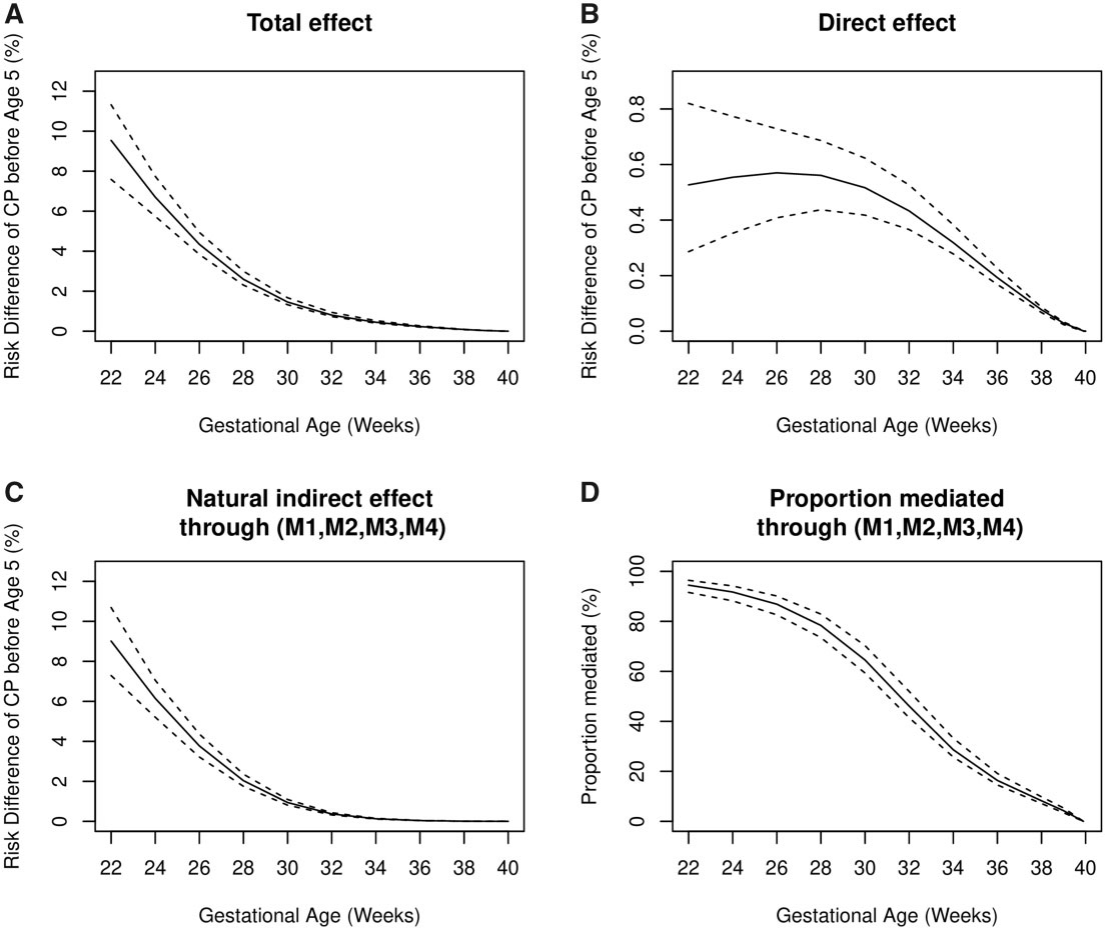
Mediation analysis for the association between gestational age and cerebral palsy (CP), Part I. (A) Total effect of gestational age on CP. (B) Direct effect of gestational age on CP. (C) Natural indirect effect mediated through all pathways involving M1, M2, M3 and M4 jointly. (D) Proportion mediated through all pathways involving M1, M2, M3 and M4 jointly. Solid lines represent risk difference in (A)–(C) and proportion mediated in (D), before 5 years of age. Dashed lines represent pointwise 95% CIs. M1=asphyxia; M2=respiratory-related diseases; M3=infection-/inflammatory-related diseases; M4=neurological-related diseases.

**Figure 2–2:**
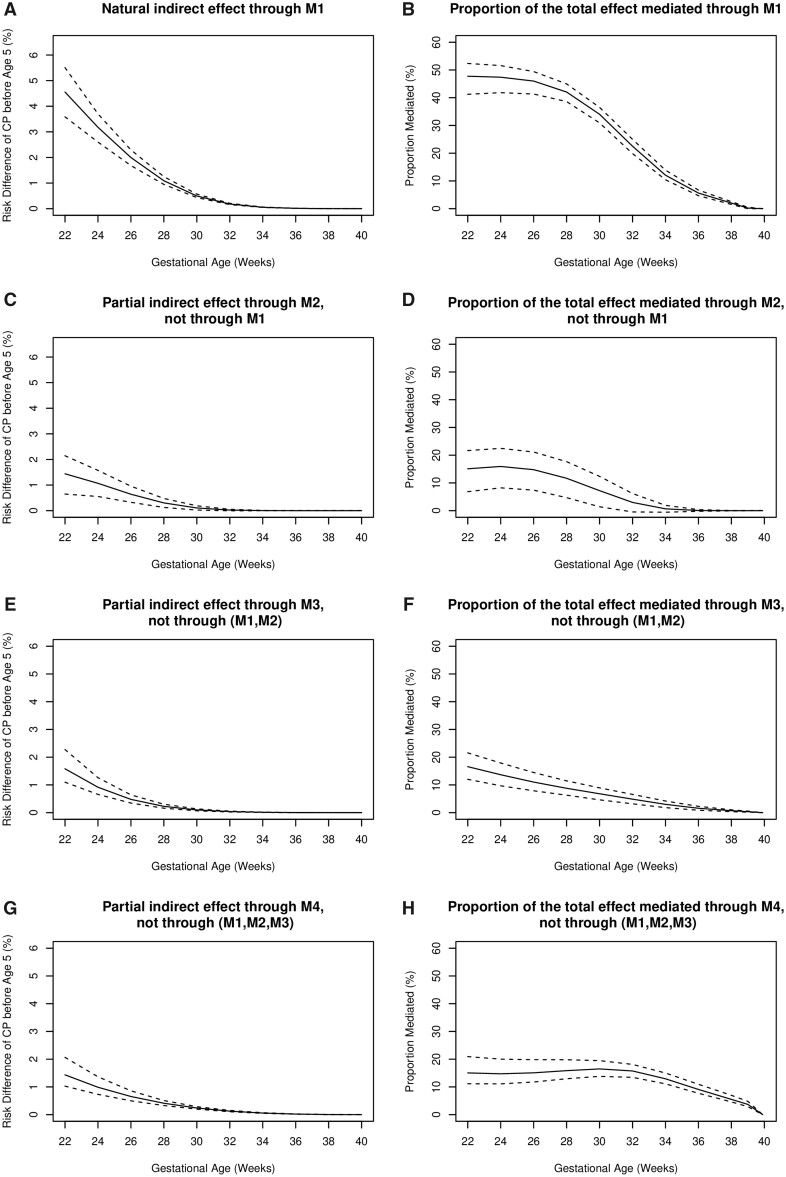
Mediation analysis for the association between gestational age and cerebral palsy (CP), Part II. (A) Natural indirect effect mediated through all pathways involving M1. (B) Proportion of the total effect mediated through all pathways involving M1. (C) Partial indirect effect mediated through all pathways involving M2, but not through M1. (D) Proportion of the total effect mediated through all pathways involving M2, but not through M1. (E) Partial indirect effect mediated through all pathways involving M3, but not through M1 or M2. (F) Proportion of the total effect mediated through all pathways involving M3, but not through M1 or M2. (G) Partial indirect effect mediated through the pathway involving M4, but not through M1, M2 or M3. (H) Proportion of the total effect mediated through the pathway involving M4, but not through M1, M2 or M3. Solid lines represent risk difference in (A), (C), (E) and (G), and proportion mediated in (B), (D), (F) and (H), before 5 years of age. Dashed lines represent pointwise 95% CIs. M1=asphyxia; M2=respiratory-related diseases; M3=infection-/inflammatory-related diseases; M4=neurological-related diseases.

## Discussion

In this nationwide population-based study of singleton children born at 22–40 gestational weeks, decreasing gestational age was, as expected, associated with increasing risk of CP. Neonatal morbidity explained 91.7% of the higher risk of CP at 24 vs 40 weeks; this proportion decreased with increasing gestational age. Pathways involving asphyxia were the main mediating pathways from 22 to 34 weeks, whereas the pathway involving only neurological-related neonatal diseases played the leading mediating role from 34 weeks onwards.

Preterm birth has been associated with higher risk of CP.^4^^,^^10^^,^^27^ Our findings corroborated previous findings by introducing survival analysis with extensive confounding adjustment and assessing the dose–response relationships of CP subtypes with gestational age. Compared with other confounders, mode of delivery and major malformation had notable confounding effects. This could potentially be due to these two factors reflecting intrauterine distress and malformation development in or outside the nervous system, which causes fetuses to be delivered early and to develop CP later.^28–30^ We choose to present these results using both relative risk scale (HR) and absolute risk scale (risk difference), to provide a comprehensive picture of the studied associations and related implications.

A two-hit theory has been proposed as the mechanisms of CP development: intrauterine factors leading to preterm delivery are the first hit, and neonatal morbidity leading to impaired neurodevelopment and subsequent CP is the second hit.^31^ With causal mediation analysis, our study contributes to a better understanding of the associations between gestational age, neonatal morbidity and CP. Pathways involving asphyxia, which may have been preceded by fetal compromise, explained a large proportion of the increased risk of CP, particularly at a very short gestational age. From 34 weeks onwards, neurological-related diseases played the leading mediating role. This result could possibly be attributed to a higher risk of CP in relation to neurological morbidity (such as intracranial haemorrhage) in late preterm infants than in very or moderately preterm infants.^27^ CP is associated with respiratory distress syndrome and sepsis in preterm children.^6^^,^^7^^,^^32^ We found that the association between gestational age and CP was to a lesser extent explained by pathways through respiratory-related diseases and infection-/inflammatory-related diseases. As gestational age increased, the proportion of the increased risk of CP explained by neonatal morbidity decreased. In preterm infants, this means that the proportion directly explained by gestational age increased with gestational age. Still, the direct effect of gestational age, an absolute measure, decreased as gestational age increased. Further research is needed to demonstrate whether other factors explain the risk of CP in infants approaching term gestation.

Less is known about the association between gestational age and CP subtypes while taking the effect of multiple confounders into consideration.^4^ Our findings showed inverse dose–response relationships between gestational age and all CP subtypes, where the highest risk was observed for spastic diplegia, the subtype that usually co-occurs with white-matter injury of prematurity.^1^

This study has several strengths. The nationwide population-based study design, together with the prospectively collected data from high-quality registries, largely precludes the possibility of selection and information bias. The large sample size of the study population with a long follow-up yielded sufficient statistical power for survival analysis of CP, a rare outcome, and allowed exploration of potential pathways through multiple mediators. We applied flexible mediation analysis with four sequential mediators and, for the first time, reported the variation in causal effects across the entire range of the exposure. This, together with the use of absolute effect measures, quantified the public health implications and provided better insight for clinicians and parents who might be concerned about the child’s absolute risk of developing CP.

Some limitations deserve consideration. The interpretation of causal mediation analysis relies on several assumptions, including no unmeasured confounding of the exposure–mediator, mediator–outcome and exposure–outcome relationships and no mediator–outcome confounder that is affected by the exposure.^33^ Like all causal assumptions, these are fundamentally untestable and must be judged by using subject-matter knowledge. In our study, we have controlled for an extensive set of known, suspected or potential confounders. However, the associations may also be confounded by maternal minor disorders before and during pregnancy and (low) socio-economic status,^5^ which we did not control for. Hence, the observed results must be interpreted with some caution. Although the temporal ordering of the mediators was decided based on a priori assumptions and previous literature, we cannot be certain of the temporal development of different neonatal diseases. We included information about the most important neonatal risk factors for CP, but our results do not imply an absence of other potential mediators. For example, we lacked specific information on postnatal events such as neonatal resuscitation, breastfeeding, skin-to-skin contact and postnatal growth. Other neurological-related diseases, such as hypoxic ischaemic encephalopathy, affected only a few preterm infants, which made them less relevant to be included as mediators. Although we applied a strategy to classify the neonatal diseases by organ system/aetiology, there is still a possibility of misclassification because one disease may have different causes. As the data of outcome were obtained from the Patient Register, we could only capture the diagnosis of CP, not the onset or severity of CP. Lastly, self-reported height may be influenced by response bias but should be non-differential across gestational age.

Understanding the mechanisms underlying the association between preterm birth and CP may facilitate the improvement of prevention and treatment of this disorder. Our findings suggest that interventions to prevent neonatal morbidity may have great benefits in the prevention of CP in preterm infants. Convulsion conferred the highest risk of CP among all studied neonatal diseases. There is a need to develop better modalities to detect its underlying causes, aimed at reducing its risk so as to minimize the risk of CP, especially in preterm births.^34^

Decreasing gestational age is associated with increasing risks of any and different subtypes of CP in singleton children born at 22–40 weeks. Neonatal morbidity may be responsible for a substantial proportion of the increased risk of CP associated with gestational age, but the magnitude declines as gestational age increases.

## Supplementary data

Supplementary data are available at *IJE* online.

## Ethics approval

This study was approved by the Regional Ethical Review Board in Stockholm, Sweden (No. 2018/20–31/5).

## Funding

This work was supported by the Swedish Research Council for Health, Working Life and Welfare [grant 2017–00134 to S.C.], the National Institute of Mental Health [grant R21MH120824 to E.V.] and an unrestricted grant from Karolinska Institutet [grant 2368/10–221 to S.C.]. No funding bodies had any role in the study design, data collection and analysis, decision to publish or preparation of the manuscript.

## Data availability

The data underlying this article were obtained from the Swedish National Board of Health and Welfare and Statistics Sweden and cannot be shared publicly according to the Swedish laws. Researchers can apply for access to these data through the Swedish National Board of Health and Welfare and Statistics Sweden after obtaining an ethics approval from a regional ethical review board.

## Author contributions

Concept and design: R.C., S.C.; acquisition of data: S.C.; analysis and interpretation of data: all authors; drafting of the manuscript: R.C.; critical revision of the manuscript for important intellectual content: all authors; final approval of the version to be published: all authors; agreement to be accountable for all aspects of the work thereby ensuring that questions related to the accuracy or integrity of any part of the work are appropriately investigated and resolved: all authors.

## Conflict of interest

None declared.

## Supplementary Material

dyab131_Supplementary_Data
