# Supplementary material for: Impact of gestational age on risk of cerebral palsy: unravelling the role of neonatal morbidity
Source: Int J Epidemiol. 2021 Jun 28;50(6):1852–63. doi: 10.1093/ije/dyab131 (PMC8743109; doi:10.1093/ije/dyab131)
Supplement: dyab131_Supplementary_Data [file dyab131_supplementary_data.docx]

**Supplementary material**

Impact of gestational age on risk of cerebral palsy: Unravelling the role of neonatal morbidity

**Supplementary Methods 1.** Swedish National Registries.

**Supplementary Methods 2.** Causal mediation analysis with four sequential mediators and survival outcome

**Supplementary Table S1.** ICD codes of maternal and child diseases

**Supplementary Table S2.** Maternal characteristics and risk of cerebral palsy in children born at 22–40 weeks in Sweden during 1998–2016

**Supplementary Table S3.** Distribution of maternal, pregnancy, and newborn characteristics across gestational age categories in children born at 22–40 weeks in Sweden during 1998–2016

**Supplementary Table S4.** Gestational age and risk of cerebral palsy in children born at 22–40 weeks in Sweden during 1998–2016, comparison between different confounders adjustment

**Supplementary Table S5.** Gestational age and risk of subtypes of cerebral palsy in children born at 22–40 weeks in Sweden during 1998–2016

**Supplementary Table S6.** Gestational age and neonatal morbidity (potential mediators) in children born at 22–40 weeks in Sweden during 1998–2016

**Supplementary Table S7**. Gestational age and risk of cerebral palsy before 5 years of age in children born at 22-40 weeks in Sweden during 1998-2016

**Supplementary Methods 1. Swedish National Registries**

The Medical Birth Register includes information about over 98% of deliveries in Sweden.^1^ The Patient Register was used to obtain information about diagnosis of CP, and, in addition to the Medical Birth Register, to obtain information about maternal diseases, malformations, and neonatal morbidity.^2^ Diagnoses were coded according to the Swedish version of International Classification of Diseases, tenth revision (ICD-10). The Cause of Death Register was used to retrieve information on the date of death.^3^ Information about mothers’ educational level and dates of emigration in children was retrieved from the Education Register and Total Population Register, respectively.^4, 5^ The Multi-Generation Register was used to identify children who were siblings.^6^

**Supplementary Methods 2. Causal mediation analysis with four sequential mediators and survival outcome**

Let $X$ be the exposure (gestational age) and let $Y$ be the outcome (indicator of whether cerebral palsy occurs before 5 years of age). Let $M_{1}, M_{2},M_{3},M_{4}$ be the four mediators (asphyxia, respiratory-related diseases, infection-/inflammatory-related diseases, and neurological-related diseases). We define the following nested counterfactuals/interventions.^7, 8^ Let

$$M_{1}\left( x^{'} \right)$$

be the counterfactual outcome of $M_{1}$ under the intervention **1** defined as: for each subject, $M_{1}$ is set to the value it would have if $X$ is set to $x^{'}$. Let

$$M_{2}\left( x^{''},M_{1}\left( x^{'} \right) \right)$$

be the counterfactual outcome of $M_{2}$ under the intervention **2** defined as: for each subject, $M_{2}$ is set to the value it would have if $X$ is set $x^{''}$ and intervention **1** is simultaneously performed. Let

$$M_{3}\left( x^{'''},M_{1}\left( x^{'} \right),M_{2}\left( x^{''},M_{1}\left( x^{'} \right) \right) \right)$$

be the counterfactual outcome of $M_{3}$ under the intervention **3** defined as: for each subject, $M_{3}$ is set to the value it would have if $X$ is set to $x^{'''}$ and interventions **1** and **2** are simultaneously performed. Let

$$M_{4}\left( x^{''''},M_{1}\left( x^{'} \right),M_{2}\left( x^{''},M_{1}\left( x^{'} \right) \right) ,M_{3}\left( x^{'''},M_{1}\left( x^{'} \right),M_{2}\left( x^{''},M_{1}\left( x^{'} \right) \right) \right) \right)$$

be the counterfactual outcome of $M_{4}$ under the intervention **4** defined as: for each subject, $M_{4}$ is set to the value it would have if $X$ is set to $x^{''''}$ and interventions **1**, **2** and **3** are simultaneously performed. Finally, let

$$Y\left( x,M_{1}\left( x^{'} \right), M_{2}\left( x^{''},M_{1}\left( x^{'} \right) \right),M_{3}\left( x^{'''},M_{1}\left( x^{'} \right),M_{2}\left( x^{''},M_{1}\left( x^{'} \right) \right) \right),M_{4}\left( x^{''''},M_{1}\left( x^{'} \right),M_{2}\left( x^{''},M_{1}\left( x^{'} \right) \right) ,M_{3}\left( x^{'''},M_{1}\left( x^{'} \right),M_{2}\left( x^{''},M_{1}\left( x^{'} \right) \right) \right) \right) \right)$$

be the counterfactual value of $Y$ under the intervention **4** defined as : for each subject, $Y$ is set to the value it would have if $X$ were set to $x$ and intervention **1**, **2**, and **3** are simultaneously performed.

As in Steen et al^9^, we define the following causal effects.

Total effect of $X$ on $Y$:

$$TE\left( x \right)=p\left\{ Y\left( x,M_{1}\left( x \right), M_{2}\left( x,M_{1}\left( x \right) \right),M_{3}\left( x,M_{1}\left( x \right),M_{2}\left( x,M_{1}\left( x \right) \right) \right), M_{4}\left( x,M_{1}\left( x \right),M_{2}\left( x,M_{1}\left( x \right) \right) ,M_{3}\left( x,M_{1}\left( x \right),M_{2}\left( x,M_{1}\left( x \right) \right) \right) \right) \right)=1 \right\}-p\left\{ Y\left( 40,M_{1}\left( 40 \right), M_{2}\left( 40,M_{1}\left( 40 \right) \right),M_{3}\left( 40,M_{1}\left( 40 \right),M_{2}\left( 40,M_{1}\left( 40 \right) \right) \right),M_{4}\left( 40,M_{1}\left( 40 \right),M_{2}\left( 40,M_{1}\left( 40 \right) \right) ,M_{3}\left( 40,M_{1}\left( 40 \right),M_{2}\left( 40,M_{1}\left( 40 \right) \right) \right) \right) \right)=1 \right\}$$

Direct effect of $X$ on $Y$:

$$DE\left( x \right)= p\left\{ Y\left( x,M_{1}\left( 40 \right), M_{2}\left( 40,M_{1}\left( 40 \right) \right),M_{3}\left( 40,M_{1}\left( 40 \right),M_{2}\left( 40,M_{1}\left( 40 \right) \right) \right),M_{4}\left( 40,M_{1}\left( 40 \right),M_{2}\left( 40,M_{1}\left( 40 \right) \right) ,M_{3}\left( 40,M_{1}\left( 40 \right),M_{2}\left( 40,M_{1}\left( 40 \right) \right) \right) \right) \right)=1 \right\}-p\left\{ Y\left( 40,M_{1}\left( 40 \right), M_{2}\left( 40,M_{1}\left( 40 \right) \right),M_{3}\left( 40,M_{1}\left( 40 \right),M_{2}\left( 40,M_{1}\left( 40 \right) \right) \right),M_{4}\left( 40,M_{1}\left( 40 \right),M_{2}\left( 40,M_{1}\left( 40 \right) \right) ,M_{3}\left( 40,M_{1}\left( 40 \right),M_{2}\left( 40,M_{1}\left( 40 \right) \right) \right) \right) \right)=1 \right\}$$

Natural indirect effect mediated through $(M_{1},M_{2},M_{3}, M_{4})$:

$$IE\left( x;M_{1},M_{2},M_{3},M_{4} \right)=p\left\{ Y\left( x,M_{1}\left( x \right), M_{2}\left( x,M_{1}\left( x \right) \right),M_{3}\left( x,M_{1}\left( x \right),M_{2}\left( x,M_{1}\left( x \right) \right) \right),M_{4}\left( x,M_{1}\left( x \right),M_{2}\left( x,M_{1}\left( x \right) \right) ,M_{3}\left( x,M_{1}\left( x \right),M_{2}\left( x,M_{1}\left( x \right) \right) \right) \right) \right)=1 \right\}-p\left\{ Y\left( x,M_{1}\left( 40 \right), M_{2}\left( 40,M_{1}\left( 40 \right) \right),M_{3}\left( 40,M_{1}\left( 40 \right),M_{2}\left( 40,M_{1}\left( 40 \right) \right) \right),M_{4}\left( 40,M_{1}\left( 40 \right),M_{2}\left( 40,M_{1}\left( 40 \right) \right) ,M_{3}\left( 40,M_{1}\left( 40 \right),M_{2}\left( 40,M_{1}\left( 40 \right) \right) \right) \right) \right)=1 \right\}$$

Natural indirect effect mediated through $M_{1}$:

$$IE\left( x;M_{1} \right)=p\left\{ Y\left( x,M_{1}\left( x \right), M_{2}\left( x,M_{1}\left( x \right) \right),M_{3}\left( x,M_{1}\left( x \right),M_{2}\left( x,M_{1}\left( x \right) \right) \right),M_{4}\left( x,M_{1}\left( x \right),M_{2}\left( x,M_{1}\left( x \right) \right) ,M_{3}\left( x,M_{1}\left( x \right),M_{2}\left( x,M_{1}\left( x \right) \right) \right) \right) \right)=1 \right\}-p\left\{ Y\left( x,M_{1}\left( 40 \right), M_{2}\left( x,M_{1}\left( 40 \right) \right),M_{3}\left( x,M_{1}\left( 40 \right),M_{2}\left( x,M_{1}\left( 40 \right) \right) \right),M_{4}\left( x,M_{1}\left( 40 \right),M_{2}\left( x,M_{1}\left( 40 \right) \right) ,M_{3}\left( x,M_{1}\left( 40 \right),M_{2}\left( x,M_{1}\left( 40 \right) \right) \right) \right) \right)=1 \right\}$$

Partial indirect effect mediated through $M_{2}$, not through $M_{1}$:

$$IE\left( x;M_{2} \right)=p\left\{ Y\left( x,M_{1}\left( 40 \right), M_{2}\left( x,M_{1}\left( 40 \right) \right),M_{3}\left( x,M_{1}\left( 40 \right),M_{2}\left( x,M_{1}\left( 40 \right) \right) \right),M_{4}\left( x,M_{1}\left( 40 \right),M_{2}\left( x,M_{1}\left( 40 \right) \right) ,M_{3}\left( x,M_{1}\left( 40 \right),M_{2}\left( x,M_{1}\left( 40 \right) \right) \right) \right) \right)=1 \right\}-p\left\{ Y\left( x,M_{1}\left( 40 \right), M_{2}\left( 40,M_{1}\left( 40 \right) \right),M_{3}\left( x,M_{1}\left( 40 \right),M_{2}\left( 40,M_{1}\left( 40 \right) \right) \right),M_{4}\left( x,M_{1}\left( 40 \right),M_{2}\left( 40,M_{1}\left( 40 \right) \right) ,M_{3}\left( x,M_{1}\left( 40 \right),M_{2}\left( 40,M_{1}\left( 40 \right) \right) \right) \right) \right)=1 \right\}$$

Partial indirect effect mediated through $M_{3}$, not through ${(M}_{1},M_{2})$:

$$IE\left( x;M_{3} \right)=p\left\{ Y\left( x,M_{1}\left( 40 \right), M_{2}\left( 40,M_{1}\left( 40 \right) \right),M_{3}\left( x,M_{1}\left( 40 \right),M_{2}\left( 40,M_{1}\left( 40 \right) \right) \right),M_{4}\left( x,M_{1}\left( 40 \right),M_{2}\left( 40,M_{1}\left( 40 \right) \right) ,M_{3}\left( x,M_{1}\left( 40 \right),M_{2}\left( 40,M_{1}\left( 40 \right) \right) \right) \right) \right)=1 \right\}-p\left\{ Y\left( x,M_{1}\left( 40 \right), M_{2}\left( 40,M_{1}\left( 40 \right) \right),M_{3}\left( 40,M_{1}\left( 40 \right),M_{2}\left( 40,M_{1}\left( 40 \right) \right) \right),M_{4}\left( x,M_{1}\left( 40 \right),M_{2}\left( 40,M_{1}\left( 40 \right) \right) ,M_{3}\left( 40,M_{1}\left( 40 \right),M_{2}\left( 40,M_{1}\left( 40 \right) \right) \right) \right) \right)=1 \right\}$$

Partial indirect effect mediated through $M_{4}$, not through ${(M}_{1}, M_{2},M_{3})$:

$$IE\left( x;M_{4} \right)=p\left\{ Y\left( x,M_{1}\left( 40 \right), M_{2}\left( 40,M_{1}\left( 40 \right) \right),M_{3}\left( 40,M_{1}\left( 40 \right),M_{2}\left( 40,M_{1}\left( 40 \right) \right) \right),M_{4}\left( x,M_{1}\left( 40 \right),M_{2}\left( 40,M_{1}\left( 40 \right) \right) ,M_{3}\left( 40,M_{1}\left( 40 \right),M_{2}\left( 40,M_{1}\left( 40 \right) \right) \right) \right) \right)=1 \right\}-p\left\{ Y\left( x,M_{1}\left( 40 \right), M_{2}\left( 40,M_{1}\left( 40 \right) \right),M_{3}\left( 40,M_{1}\left( 40 \right),M_{2}\left( 40,M_{1}\left( 40 \right) \right) \right),M_{4}\left( 40,M_{1}\left( 40 \right),M_{2}\left( 40,M_{1}\left( 40 \right) \right) ,M_{3}\left( 40,M_{1}\left( 40 \right),M_{2}\left( 40,M_{1}\left( 40 \right) \right) \right) \right) \right)=1 \right\}$$

From these definitions of causal effects, we have that

$$TE\left( x \right)=DE\left( x \right)+IE(x;M_{1},M_{2},M_{3},M_{4})$$

and

$$IE\left( x;M_{1},M_{2},M_{3},M_{4} \right)= IE\left( x;M_{1} \right)+IE\left( x;M_{2} \right)+IE\left( x;M_{3} \right)+IE\left( x;M_{4} \right)$$

In the causal effects above, pathways are “blocked” by counterfactual interventions. To understand the idea, consider the direct effect $DE\left( x \right)$, which is a contrast between two probabilities. In the first probability $X$ is set to $x$, and in the second probability $X$ is set to 40. However, in both probabilities, $M_{1}$ is set to whatever value it would have had, had $X$ been set to 40. Thus, for each subject the value of $M_{1}$ is the same in the two probabilities, so that the path through $M_{1}$ is “blocked”. In a similar way, the paths through $M_{2}$, $M_{3}$ and $M_{4}$ are blocked as well in the two probabilities.

Let $Z$ be the set of measured confounders, as describes in the main text. It follows from Steen et al^9^ that, under the directed acyclic graph in Figure 1, the counterfactual probability

$$p\left\{ Y\left( x,M_{1}\left( x^{'} \right), M_{2}\left( x^{''},M_{1}\left( x^{'} \right) \right),M_{3}\left( x^{'''},M_{1}\left( x^{'} \right),M_{2}\left( x^{''},M_{1}\left( x^{'} \right) \right) \right),M_{4}\left( x^{''''},M_{1}\left( x^{'} \right),M_{2}\left( x^{''},M_{1}\left( x^{'} \right) \right) ,M_{3}\left( x^{'''},M_{1}\left( x^{'} \right),M_{2}\left( x^{''},M_{1}\left( x^{'} \right) \right) \right) \right) \right)=1 \right\} (1)$$

is identifiable, and equal to

$$\sum_{{z,m}_{1},m_{2},m_{3},m_{4}} p(Y=1|z,x,m_{1},m_{2},m_{3},m_{4})p(m_{1}|z,x')p(m_{2}|{z,x}^{''},m_{1})p(m_{3}|z,x^{'''},m_{1},m_{2})p(m_{4}|z,x^{''''},m_{1},m_{2},m_{3})p(z) (2)$$

All causal effects above are contrasts between counterfactual probabilities on the form of (1); thus, all causal effects above are identifiable as well.

To estimate the counterfactual probability (1) we fitted the logistic regression models

$$logit\left\{ p\left( M_{1} | Z,X \right) \right\}=g_{1}\left( Z,X;\beta_{1} \right)$$

$$logit\left\{ p\left( M_{2} | Z,X,M_{1} \right) \right\}=g_{2}\left( Z,X,M_{2};\beta_{2} \right)$$

$$logit\left\{ p\left( M_{3} | Z,X,M_{1},M_{2} \right) \right\}=g_{3}\left( Z,X,M_{1},M_{2};\beta_{3} \right)$$

$$logit\left\{ p\left( M_{4} | Z,X,M_{1},M_{2},M_{3} \right) \right\}=g_{4}\left( Z,X,M_{1},M_{2},M_{3};\beta_{4} \right)$$

and the Cox proportional hazards regression model

$$\lambda\left( t | Z,X,M_{1},M_{2},M_{3},M_{4} \right)=\lambda_{0}\left( t \right)\exp\left\{ g_{5}\left( Z,X,M_{1},M_{2},M_{3},M_{4};\beta_{5} \right) \right\},$$

where $\lambda\left( t | Z,X,M_{1},M_{2},M_{3},M_{4} \right)$ is the conditional hazard function in the distribution of time to cerebral palsy. In these models, the regressions functions $g_{1},g_{2},g_{3},g_{4},g_{5}$ were assumed to be linear functions of the included variables, except for $X$, which was modeled in all functions with a cubic spline, with three degrees of freedom. The fitted logistic models were used to estimate $p(m_{1}|z,x')$, $p(m_{2}|{z,x}^{''},m_{1})$ , $p(m_{3}|z,x^{'''},m_{1},m_{2})$, and $p\left( m_{4} | z,x^{''''},m_{1},m_{2},m_{3} \right)$ for each level of $(z,x^{'},x^{''},x^{'''},{x^{''''},m}_{1},m_{2},m_{3,}m_{4})$, and the fitted Cox proportional hazards regression model was used, together with Breslow’s estimator of the baseline cumulative hazard function, to estimate $p(Y=1|z,x,m_{1},m_{2},m_{3},m_{4})$, for each level of $(z,x, m_{1},m_{2},m_{3},m_{4})$. Finally, these estimates were plugged into the expression in (2) to produce an estimate of the counterfactual probability (1).

**References**

1. The Swedish Medical Birth Register. <https://www.socialstyrelsen.se/en/statistics-and-data/registers/register-information/the-swedish-medical-birth-register/> (18 September 2020, date last accessed).

2. Ludvigsson JF, Andersson E, Ekbom A, et al. External review and validation of the Swedish national inpatient register. BMC Public Health. 2011;11:450.

3. Brooke HL, Talback M, Hornblad J, et al. The Swedish cause of death register. Eur J Epidemiol. 2017;32(9):765-73.

4. Statistics Sweden. Register on Participation in Education. <https://www.scb.se/uf0507-en>. (18 September 2020, date last accessed).

5. Ludvigsson JF, Almqvist C, Bonamy AK, et al. Registers of the Swedish total population and their use in medical research. Eur J Epidemiol. 2016;31(2):125-36.

6. Ekbom A. The Swedish Multi-generation Register. Methods in Biobanking. 675. Totowa, NJ: Humana Press; 2011.

7. Rubin DB. Estimating causal effects of treatments in randomized and nonrandomized studies. J Educ Psychol. 1974;66(5):688-701.

8. Pearl J. Causality: Cambridge University Press 2009.

9. Steen J, Loeys T, Moerkerke B, Vansteelandt S. Flexible Mediation Analysis With Multiple Mediators. Am J Epidemiol. 2017;186(2):184-93.

| **Supplementary Table S1. ICD codes of maternal and child diseases** | |
| --- | --- |
| **Diagnoses** | **ICD-10 codes** |
| **Mother^a^** |  |
| Diabetic diseases |  |
| Pregestational diabetes | E10–E14, O24.0–O24.3 |
| Gestational diabetes | O24.4 |
| Hypertensive diseases |  |
| Pregestational hypertension | O10–O11, I10–I15 |
| Preeclampsia | O14–O15 |
| Chorioamnionitis | O41.1 |
| Infectious diseases during pregnancy | O98 |
| Placental abruption | O45 |
| **Newborn** |  |
| Major malformation^b^ | Q00–Q99, excluding^c^ Q17.0, Q17.5, Q18.0, Q18.1, Q25.0, Q27.0, Q31.5, Q32.0, Q38.1, Q52.3, Q53.0–Q53.9, Q65.0–Q65.9, Q66.5–Q66.9, Q69.0, Q69.9, Q70.3, Q76.0, Q79.9, Q82.5, Q82.9 |
| **Potential mediators (Neonatal diseases)^d^** |  |
| Asphyxia |  |
| *Intrapartum hypoxia* | P20.1 |
| *Birth asphyxia* | P21 |
| Respiratory-related diseases |  |
| *Respiratory distress syndrome* | P22.0 |
| *Bronchopulmonary dysplasia* | P27.1 |
| Infection-/inflammatory-related diseases |  |
| *Sepsis* | P36.0–P36.8 |
| *Other infections specific to the perinatal period* | P38–P39 |
| *Pneumonia* | P23, J12–J18 |
| *Central nervous system inflammatory diseases* | G00–G09 |
| *Necrotizing enterocolitis* | P77 |
| Neurological-related diseases |  |
| *Convulsion* | P90 |
| *Intracranial nontraumatic haemorrhage* | P52 |
| *Periventricular leukomalacia* | P91.2 |
| **Subtypes of CP**^e^ |  |
| Spastic quadriplegic CP | G80.0 |
| Spastic diplegic CP | G80.1 |
| Spastic hemiplegic CP | G80.2 |
| Dyskinetic CP | G80.3 |
| Ataxic CP | G80.4 |
| Other or unspecified CP | G80.8–G80.9 |
| ICD, International Classification of Diseases; CP, cerebral palsy. | |
| ^a^ Information on diagnoses are obtained from the Medical Birth Register or the Patient Register (diagnosis date during pregnancy). | |
| ^b^ Information on diagnoses are obtained from the Medical Birth Register or the Patient Register (diagnosis date during 0–364 days of life). | |
| ^c^ The following ICD codes represent diagnoses of minor malformations, defined by the Swedish National Board of Health and Welfare (see https://www.socialstyrelsen.se/globalassets/sharepoint-dokument/dokument-webb/ovrigt/diagnoser-som-inte-ska-rapporteras-om-fosterskador.pdf). | |
| ^d^ Information on diagnoses are obtained from the Medical Birth Register or the Patient Register (inpatient care, admission date during 0–27 days of life). | |
| ^e^ Information on diagnoses are obtained from the Patient Register. | |

| **Supplementary Table S2. Maternal characteristics and risk of cerebral palsy in children born at 22–40 weeks in Sweden during 1998–2016** | | | | | |
| --- | --- | --- | --- | --- | --- |
| **Maternal characteristics** | **No. of children (%)** | **Person years** | **Cerebral Palsy** | | |
|  |  |  | **No. of cases** | **Rate^a^** | **Hazard Ratio (95%CI)^b^** |
| Total | 1 402 240 | 13 074 532 | 3245 | 2.48 |  |
| Age at childbirth (years) |  |  |  |  |  |
| <20 | 22 416 (1.60) | 227 172 | 73 | 3.21 | 1.40 (1.09–1.79) |
| 20–24 | 183 695 (13.10) | 1 733 868 | 452 | 2.61 | 1.14 (1.02–1.28) |
| 25–29 | 431 681 (30.79) | 4 094 223 | 932 | 2.28 | Ref. |
| 30–34 | 478 708 (34.14) | 4 478 329 | 1074 | 2.40 | 1.03 (0.95–1.13) |
| ≥35 | 285 740 (20.38) | 2 540 939 | 714 | 2.81 | 1.18 (1.07–1.30) |
| Parity |  |  |  |  |  |
| 1 | 593 351 (42.31) | 5 559 787 | 1564 | 2.81 | Ref. |
| 2 | 534 695 (38.13) | 4 959 657 | 1005 | 2.03 | 0.72 (0.66–0.78) |
| 3 | 191 984 (13.69) | 1 788 275 | 452 | 2.53 | 0.90 (0.81–1.00) |
| ≥4 | 82 210 (5.86) | 766 813 | 224 | 2.92 | 1.05 (0.91–1.22) |
| Educational level (years) |  |  |  |  |  |
| ≤9 | 124 441 (8.87) | 1 089 396 | 367 | 3.37 | 1.58 (1.40–1.78) |
| 10–11 | 176 239 (12.57) | 1 985 879 | 549 | 2.76 | 1.50 (1.35–1.66) |
| 12 | 367 287 (26.19) | 3 354 773 | 862 | 2.57 | 1.22 (1.12–1.34) |
| 13–14 | 195 137 (13.92) | 1 871 007 | 412 | 2.20 | 1.09 (0.97–1.22) |
| ≥15 | 528 615 (37.70) | 4 723 180 | 1018 | 2.16 | Ref. |
| Missing | 10 521 (0.75) | 50 297 | 37 | 7.36 |  |
| Country of birth |  |  |  |  |  |
| Non-Nordic | 290 017 (20.68) | 2 358 270 | 659 | 2.79 | 1.06 (0.97–1.15) |
| Nordic | 1 110 733 (79.21) | 10 709 351 | 2579 | 2.41 | Ref. |
| Missing | 1490 (0.11) | 6911 | 7 | 10.13 |  |
| Smoking during pregnancy |  |  |  |  |  |
| No | 1 220 698 (87.05) | 11 177 305 | 2624 | 2.35 | Ref. |
| Yes | 117 740 (8.40) | 1 245 778 | 381 | 3.06 | 1.42 (1.28–1.59) |
| Missing | 63 802 (4.55) | 651 449 | 240 | 3.68 |  |
| Height (cm) |  |  |  |  |  |
| <160 | 205 270 (14.64) | 1 859 494 | 534 | 2.87 | 1.18 (1.06–1.31) |
| 160–164 | 363 824 (25.95) | 3 387 908 | 844 | 2.49 | 1.03 (0.93–1.13) |
| 165–169 | 397 957 (28.38) | 3 750 210 | 898 | 2.39 | Ref. |
| ≥170 | 412 892 (29.45) | 3 882 766 | 882 | 2.27 | 0.94 (0.86–1.04) |
| Missing | 22 297 (1.59) | 194 154 | 87 | 4.48 |  |
| Early pregnancy BMI |  |  |  |  |  |
| <18.5 | 34 269 (2.44) | 309 548 | 92 | 2.97 | 1.35 (1.09–1.67) |
| 18.5–24.9 | 784 379 (55.94) | 7 298 503 | 1603 | 2.20 | Ref. |
| 25.0–29.9 | 312 090 (22.26) | 2 843 411 | 766 | 2.69 | 1.22 (1.12–1.33) |
| ≥30.0 | 145 615 (10.38) | 1 262 038 | 424 | 3.36 | 1.45 (1.30–1.62) |
| Missing | 125 887 (8.98) | 1 361 032 | 360 | 2.65 |  |
| ^a^ Rate is calculated as number of cases per 10 000 person-years. | | | | | |
| ^b^ Unadjusted model. | | | | | |

**Supplementary Table S3. Distribution of maternal, pregnancy, and newborn characteristics across gestational age categories in children born at 22–40 weeks in Sweden during 1998–2016**

| **Characteristics** | **No. of children (%)** | **Gestational age (weeks, N [%])** | | | | | | |
| --- | --- | --- | --- | --- | --- | --- | --- | --- |
|  |  | **22–24** | **25–27** | **28–31** | **32–34** | **35–36** | **37–38** | **39–40** |
| Total^a^ | 1 402 240 (100.0) | 832 (0.1) | 2744 (0.2) | 8063 (0.6) | 21 975 (1.6) | 56 011 (4.0) | 346 859 (24.7) | 965 756 (68.9) |
| **Maternal** |  |  |  |  |  |  |  |  |
| **Age at childbirth (years)** |  |  |  |  |  |  |  |  |
| <20 | 22 416 (1.6) | 36 (4.3) | 63 (2.3) | 187 (2.3) | 468 (2.1) | 1114 (2.0) | 5515 (1.6) | 15 033 (1.6) |
| 20–24 | 183 695 (13.1) | 124 (14.9) | 386 (14.1) | 1072 (13.3) | 3144 (14.3) | 7952 (14.2) | 43 283 (12.5) | 127 734 (13.2) |
| 25–29 | 431 681 (30.8) | 231 (27.8) | 744 (27.1) | 2309 (28.6) | 6591 (30.0) | 17 339 (31.0) | 102 090 (29.4) | 302 377 (31.3) |
| 30–34 | 478 708 (34.1) | 212 (25.5) | 880 (32.1) | 2560 (31.7) | 6961 (31.7) | 17 783 (31.7) | 117 094 (33.8) | 333 218 (34.5) |
| ≥35 | 285 740 (20.4) | 229 (27.5) | 671 (24.5) | 1935 (24.0) | 4811 (21.9) | 11 823 (21.1) | 78 877 (22.7) | 187 394 (19.4) |
| **Parity** |  |  |  |  |  |  |  |  |
| 1 | 593 351 (42.3) | 483 (58.1) | 1505 (54.8) | 4543 (56.3) | 12 374 (56.3) | 29 399 (52.5) | 140 142 (40.4) | 404 905 (41.9) |
| 2 | 534 695 (38.1) | 195 (23.4) | 751 (27.4) | 2138 (26.5) | 5872 (26.7) | 16 535 (29.5) | 130 415 (37.6) | 378 789 (39.2) |
| 3 | 191 984 (13.7) | 91 (10.9) | 308 (11.2) | 872 (10.8) | 2280 (10.4) | 6398 (11.4) | 51 995 (15.0) | 130 040 (13.5) |
| ≥4 | 82 210 (5.9) | 63 (7.6) | 180 (6.6) | 510 (6.3) | 1449 (6.6) | 3679 (6.6) | 24 307 (7.0) | 52 022 (5.4) |
| **Educational level (years)** |  |  |  |  |  |  |  |  |
| ≤9 | 124 441 (8.9) | 126 (15.1) | 322 (11.7) | 922 (11.4) | 2218 (10.1) | 5579 (10.0) | 33 502 (9.7) | 81 772 (8.5) |
| 10–11 | 176 239 (12.6) | 141 (16.9) | 418 (15.2) | 1181 (14.6) | 3168 (14.4) | 7886 (14.1) | 46 828 (13.5) | 116 617 (12.1) |
| 12 | 367 287 (26.2) | 206 (24.8) | 752 (27.4) | 2120 (26.3) | 6052 (27.5) | 15 237 (27.2) | 90 958 (26.2) | 251 962 (26.1) |
| 13–14 | 195 137 (13.9) | 113 (13.6) | 396 (14.4) | 1154 (14.3) | 2898 (13.2) | 7557 (13.5) | 48 035 (13.8) | 134 984 (14.0) |
| ≥15 | 528 615 (37.7) | 233 (28.0) | 833 (30.4) | 2609 (32.4) | 7442 (33.9) | 19 386 (34.6) | 125 000 (36.0) | 373 112 (38.6) |
| Missing | 10 521 (0.8) | 13 (1.6) | 23 (0.8) | 77 (1.0) | 197 (0.9) | 366 (0.7) | 2536 (0.7) | 7309 (0.8) |
| **Country of birth** |  |  |  |  |  |  |  |  |
| Non-Nordic | 29 0017 (20.7) | 269 (32.3) | 676 (24.6) | 1698 (21.1) | 4284 (19.5) | 10 921 (19.5) | 75 191 (21.7) | 196 978 (20.4) |
| Nordic | 1 110 733 (79.2) | 559 (67.2) | 2060 (75.1) | 6348 (78.7) | 17 665 (80.4) | 45 042 (80.4) | 271 345 (78.2) | 767 714 (79.5) |
| Missing | 1490 (0.1) | 4 (0.5) | 8 (0.3) | 17 (0.2) | 26 (0.1) | 48 (0.1) | 323 (0.1) | 1064 (0.1) |
| **Smoking during pregnancy** |  |  |  |  |  |  |  |  |
| No | 1 220 698 (87.1) | 481 (57.8) | 1871 (68.2) | 6173 (76.6) | 17 643 (80.3) | 46 735 (83.4) | 297 885 (85.9) | 849 910 (88.0) |
| Yes | 117 740 (8.4) | 74 (8.9) | 295 (10.8) | 893 (11.1) | 2375 (10.8) | 5723 (10.2) | 32 192 (9.3) | 76 188 (7.9) |
| Missing | 63 802 (4.6) | 277 (33.3) | 578 (21.1) | 997 (12.4) | 1957 (8.9) | 3553 (6.3) | 16 782 (4.8) | 39 658 (4.1) |
| **Height (cm)** |  |  |  |  |  |  |  |  |
| <160 | 205 270 (14.6) | 135 (16.2) | 470 (17.1) | 1428 (17.7) | 3776 (17.2) | 9697 (17.3) | 58 990 (17.0) | 130 774 (13.5) |
| 160–164 | 363 824 (25.9) | 204 (24.5) | 715 (26.1) | 2131 (26.4) | 5958 (27.1) | 15 295 (27.3) | 93 182 (26.9) | 246 339 (25.5) |
| 165–169 | 397 957 (28.4) | 174 (20.9) | 692 (25.2) | 2191 (27.2) | 6039 (27.5) | 15 415 (27.5) | 95 752 (27.6) | 277 694 (28.8) |
| ≥170 | 412 892 (29.4) | 204 (24.5) | 635 (23.1) | 1942 (24.1) | 5505 (25.1) | 14 398 (25.7) | 93 192 (26.9) | 297 016 (30.8) |
| Missing | 22 297 (1.6) | 115 (13.8) | 232 (8.5) | 371 (4.6) | 697 (3.2) | 1206 (2.2) | 5743 (1.7) | 13 933 (1.4) |
| **Early pregnancy BMI** |  |  |  |  |  |  |  |  |
| <18.5 | 34 269 (2.4) | 15 (1.8) | 55 (2.0) | 190 (2.4) | 654 (3.0) | 1588 (2.8) | 9286 (2.7) | 22 481 (2.3) |
| 18.5–24.9 | 784 379 (55.9) | 277 (33.3) | 1121 (40.9) | 3732 (46.3) | 10 942 (49.8) | 29 034 (51.8) | 188 061 (54.2) | 551 212 (57.1) |
| 25.0–29.9 | 312 090 (22.3) | 146 (17.5) | 546 (19.9) | 1709 (21.2) | 4836 (22.0) | 12 449 (22.2) | 77 451 (22.3) | 214 953 (22.3) |
| ≥30.0 | 145 615 (10.4) | 100 (12.0) | 374 (13.6) | 1127 (14.0) | 2676 (12.2) | 6873 (12.3) | 39 478 (11.4) | 94 987 (9.8) |
| Missing | 125 887 (9.0) | 294 (35.3) | 648 (23.6) | 1305 (16.2) | 2867 (13.0) | 6067 (10.8) | 32 583 (9.4) | 82 123 (8.5) |
| **Pregnancy** |  |  |  |  |  |  |  |  |
| **Diabetic diseases** |  |  |  |  |  |  |  |  |
| No | 1 374 236 (98.0) | 818 (98.3) | 2686 (97.9) | 7747 (96.1) | 20 931 (95.2) | 53 324 (95.2) | 336 264 (96.9) | 952 466 (98.6) |
| Pregestational diabetes | 10 902 (0.8) | 5 (0.6) | 31 (1.1) | 188 (2.3) | 637 (2.9) | 1524 (2.7) | 4715 (1.4) | 3802 (0.4) |
| Gestational diabetes | 17 102 (1.2) | 9 (1.1) | 27 (1.0) | 128 (1.6) | 407 (1.9) | 1163 (2.1) | 5880 (1.7) | 9488 (1.0) |
| **Hypertensive diseases** |  |  |  |  |  |  |  |  |
| No | 1 346 935 (96.1) | 755 (90.7) | 2148 (78.3) | 5779 (71.7) | 18 103 (82.4) | 49 820 (88.9) | 329 101 (94.9) | 941 229 (97.5) |
| Pregestational hypertension | 10 925 (0.8) | 18 (2.2) | 109 (4.0) | 317 (3.9) | 501 (2.3) | 885 (1.6) | 3429 (1.0) | 5666 (0.6) |
| Preeclampsia | 44 380 (3.2) | 59 (7.1) | 487 (17.7) | 1967 (24.4) | 3371 (15.3) | 5306 (9.5) | 14 329 (4.1) | 18 861 (2.0) |
| **Chorioamnionitis** |  |  |  |  |  |  |  |  |
| No | 1 399 109 (99.8) | 661 (79.4) | 2424 (88.3) | 7701 (95.5) | 21 611 (98.3) | 55 804 (99.6) | 346 430 (99.9) | 964 478 (99.9) |
| Yes | 3131 (0.2) | 171 (20.6) | 320 (11.7) | 362 (4.5) | 364 (1.7) | 207 (0.4) | 429 (0.1) | 1278 (0.1) |
| **Infectious diseases during pregnancy** |  |  |  |  |  |  |  |  |
| No | 1 359 925 (97.0) | 749 (90.0) | 2516 (91.7) | 7615 (94.4) | 20 691 (94.2) | 53 450 (95.4) | 335 813 (96.8) | 939 091 (97.2) |
| Yes | 42 315 (3.0) | 83 (10.0) | 228 (8.3) | 448 (5.6) | 1284 (5.8) | 2561 (4.6) | 11 046 (3.2) | 26 665 (2.8) |
| **Placental abruption** |  |  |  |  |  |  |  |  |
| No | 1 396 394 (99.6) | 761 (91.5) | 2418 (88.1) | 7257 (90.0) | 20 864 (94.9) | 55 016 (98.2) | 345 652 (99.7) | 964 426 (99.9) |
| Yes | 5846 (0.4) | 71 (8.5) | 326 (11.9) | 806 (10.0) | 1111 (5.1) | 995 (1.8) | 1207 (0.3) | 1330 (0.1) |
| **Mode of delivery** |  |  |  |  |  |  |  |  |
| Noninstrumental vaginal | 1 075 533 (76.7) | 519 (62.4) | 929 (33.9) | 2626 (32.6) | 11 144 (50.7) | 37 363 (66.7) | 226 238 (65.2) | 796 714 (82.5) |
| Emergency instrumental^b^ | 177 135 (12.6) | 194 (23.3) | 818 (29.8) | 1805 (22.4) | 4581 (20.8) | 10 583 (18.9) | 39 309 (11.3) | 119 845 (12.4) |
| Elective cesarean section | 143 688 (10.3) | 113 (13.6) | 913 (33.3) | 3410 (42.3) | 5893 (26.8) | 7604 (13.6) | 78 435 (22.6) | 47 320 (4.9) |
| Unspecified cesarean section | 5884 (0.4) | 6 (0.7) | 84 (3.1) | 222 (2.8) | 357 (1.6) | 461 (0.8) | 2877 (0.8) | 1877 (0.2) |
| **Newborn** |  |  |  |  |  |  |  |  |
| **Sex** |  |  |  |  |  |  |  |  |
| Male | 706 001 (50.3) | 463 (55.6) | 1495 (54.5) | 4497 (55.8) | 12 241 (55.7) | 29 997 (53.6) | 174 825 (50.4) | 482 483 (50.0) |
| Female | 696 239 (49.7) | 369 (44.4) | 1249 (45.5) | 3566 (44.2) | 9734 (44.3) | 26 014 (46.4) | 172 034 (49.6) | 483 273 (50.0) |
| **Major malformation** |  |  |  |  |  |  |  |  |
| No | 1 339 949 (95.6) | 653 (78.5) | 2136 (77.8) | 6781 (84.1) | 19 616 (89.3) | 51 643 (92.2) | 329 311 (94.9) | 929 809 (96.3) |
| Yes | 62 291 (4.4) | 179 (21.5) | 608 (22.2) | 1282 (15.9) | 2359 (10.7) | 4368 (7.8) | 17 548 (5.1) | 35 947 (3.7) |
| **Birth weight for gestational age (percentile)** |  |  |  |  |  |  |  |  |
| <3 | 35 606 (2.5) | 77 (9.3) | 678 (24.7) | 2086 (25.9) | 2740 (12.5) | 3309 (5.9) | 9198 (2.7) | 17 518 (1.8) |
| 3 to <10 | 83 416 (5.9) | 79 (9.5) | 340 (12.4) | 1082 (13.4) | 2251 (10.2) | 3960 (7.1) | 19 434 (5.6) | 56 270 (5.8) |
| 10 to 90 | 1 134 024 (80.9) | 616 (74.0) | 1589 (57.9) | 4405 (54.6) | 14 702 (66.9) | 41 576 (74.2) | 272 378 (78.5) | 798 758 (82.7) |
| 90 to 97 | 89 638 (6.4) | 12 (1.4) | 28 (1.0) | 121 (1.5) | 883 (4.0) | 3455 (6.2) | 25 465 (7.3) | 59 674 (6.2) |
| ≥97 | 55 508 (4.0) | 13 (1.6) | 15 (0.5) | 136 (1.7) | 987 (4.5) | 3317 (5.9) | 19 312 (5.6) | 31 728 (3.3) |
| Missing | 4048 (0.3) | 35 (4.2) | 94 (3.4) | 233 (2.9) | 412 (1.9) | 394 (0.7) | 1072 (0.3) | 1808 (0.2) |
| **Calendar year of birth** |  |  |  |  |  |  |  |  |
| 1998–2002 | 312 907 (22.3) | 131 (15.7) | 628 (22.9) | 1949 (24.2) | 5154 (23.5) | 13 205 (23.6) | 77 353 (22.3) | 214 487 (22.2) |
| 2003–2007 | 363 493 (25.9) | 196 (23.6) | 698 (25.4) | 2156 (26.7) | 5886 (26.8) | 14 861 (26.5) | 93 730 (27.0) | 245 966 (25.5) |
| 2008–2012 | 399 398 (28.5) | 232 (27.9) | 765 (27.9) | 2219 (27.5) | 6106 (27.8) | 15 753 (28.1) | 98 150 (28.3) | 276 173 (28.6) |
| 2013–2016 | 326 442 (23.3) | 273 (32.8) | 653 (23.8) | 1739 (21.6) | 4829 (22.0) | 12 192 (21.8) | 77 626 (22.4) | 229 130 (23.7) |
| ^a^ Row percentage. | | | | | | | | |
| ^b^ Instrumental vaginal delivery or emergency cesarean section. | | | | | | | | |
|  | | | | | | | | |

| **Supplementary Table S4. Gestational age and risk of cerebral palsy in children born at 22–40 weeks in Sweden during 1998–2016, comparison between different confounders adjustment** | | | | | | |
| --- | --- | --- | --- | --- | --- | --- |
| **Gestational age (weeks)** | **Hazard Ratio (95%CI)** | | | | | |
|  | **Model 2^a^** | **Model 2^a^ + Mode of delivery** | **Model 2^a^ + Major malformation** | **Model 2^a^ + Mode of delivery + Major malformation** | **Model 2^a^ + Other confounders^b^** | **Model 3^c^** |
| **As continuous variable (per one week decrease)** | 1.36 (1.35–1.37) | 1.34 (1.33–1.35) | 1.33 (1.31–1.34) | 1.31 (1.29–1.32) | 1.34 (1.32–1.35) | 1.29 (1.28–1.31) |
| **As categorical variable** |  |  |  |  |  |  |
| 22–24 | 77.23 (58.01–102.81) | 69.57 (52.13–92.83) | 54.30 (40.21–73.34) | 49.78 (36.80–67.35) | 66.74 (48.72–91.42) | 47.26 (34.09–65.53) |
| 25–27 | 52.00 (43.74–61.81) | 40.43 (33.76–48.42) | 35.13 (29.20–42.26) | 28.29 (23.37–34.24) | 39.59 (32.35–48.46) | 24.18 (19.51–29.97) |
| 28–31 | 30.81 (27.05–35.09) | 24.29 (21.04–28.03) | 23.18 (20.16–26.65) | 18.81 (16.17–21.88) | 23.84 (20.25–28.05) | 16.29 (13.69–19.39) |
| 32–34 | 8.14 (7.04–9.40) | 6.93 (5.98–8.04) | 6.87 (5.93–7.96) | 5.95 (5.12–6.92) | 7.16 (6.14–8.35) | 5.53 (4.72–6.49) |
| 35–36 | 2.76 (2.37–3.21) | 2.53 (2.18–2.95) | 2.49 (2.14–2.89) | 2.29 (1.97–2.67) | 2.59 (2.22–3.02) | 2.20 (1.88–2.57) |
| 37–38 | 1.43 (1.30–1.57) | 1.32 (1.20–1.46) | 1.37 (1.25–1.51) | 1.29 (1.17–1.42) | 1.42 (1.29–1.56) | 1.30 (1.18–1.44) |
| 39–40 | Ref. | Ref. | Ref. | Ref. | Ref. | Ref. |
| ^a^ Model 2 adjusted for maternal age at childbirth, parity, educational level, country of birth, smoking during pregnancy, height, and early pregnancy BMI. | | | | | | |
| ^b^ Other confounders include diabetic diseases, hypertensive diseases, chorioamnionitis, infectious diseases during pregnancy, placental abruption, and child's sex, birth weight for gestational age, and calendar year of birth. | | | | | | |
| ^c^ Model 3 adjusted for maternal age at childbirth, parity, educational level, country of birth, smoking during pregnancy, height, early pregnancy BMI, diabetic diseases, hypertensive diseases, chorioamnionitis, infectious diseases during pregnancy, placental abruption, mode of delivery, and child's sex, major malformation, birth weight for gestational age, and calendar year of birth. | | | | | | |

| **Supplementary Table S5. Gestational age and risk of subtypes of cerebral palsy in children born at 22–40 weeks in Sweden during 1998–2016** | | | | | | | |
| --- | --- | --- | --- | --- | --- | --- | --- |
| **Exposure** | **Any CP** | **Spastic quadriplegic CP** | **Spastic diplegic CP** | **Spastic hemiplegic CP** | **Dyskinetic CP** | **Ataxic CP** | **Other or unspecified CP** |
| **No. of cases** | 3245 | 202 | 992 | 1050 | 275 | 108 | 618 |
| **Rate^a^** | 2.48 | 0.15 | 0.76 | 0.80 | 0.21 | 0.08 | 0.47 |
| **Gestational age (weeks, Hazard Ratio [95%CI]**^b^**)** |  |  |  |  |  |  |  |
| **As continuous variable (per one week decrease)** | 1.29 (1.28–1.31) | 1.20 (1.13–1.27) | 1.40 (1.38–1.43) | 1.27 (1.25–1.30) | 1.17 (1.11–1.24) | 1.20 (1.11–1.29) | 1.21 (1.17–1.25) |
| **As categorical variable** |  |  |  |  |  |  |  |
| 22–24 | 47.26 (34.09–65.53) | 8.61 (1.03–71.65) | 116.25 (71.08–190.13) | 37.42 (20.09–69.69) | 26.19 (7.35–93.29) | 33.53 (5.16–217.93) | 30.30 (14.32–64.11) |
| 25–27 | 24.18 (19.51–29.97) | 9.04 (3.54–23.05) | 72.80 (52.58–100.80) | 20.54 (13.80–30.57) | 6.86 (2.72–17.30) | 12.09 (3.86–37.89) | 9.80 (5.49–17.49) |
| 28–31 | 16.29 (13.69–19.39) | 6.94 (3.10–15.54) | 52.47 (40.07–68.72) | 12.04 (8.77–16.54) | 4.70 (2.23–9.92) | 5.51 (1.93–15.71) | 5.71 (3.58–9.12) |
| 32–34 | 5.53 (4.72–6.49) | 2.57 (1.16–5.70) | 11.58 (8.90–15.07) | 5.01 (3.79–6.61) | 3.28 (1.86–5.80) | 1.55 (0.48–5.03) | 3.50 (2.34–5.24) |
| 35–36 | 2.20 (1.88–2.57) | 1.65 (0.86–3.16) | 2.48 (1.80–3.41) | 2.38 (1.85–3.07) | 1.68 (1.00–2.81) | 1.87 (0.87–4.03) | 1.94 (1.37–2.75) |
| 37–38 | 1.30 (1.18–1.44) | 1.20 (0.80–1.81) | 1.47 (1.20–1.81) | 1.24 (1.05–1.47) | 1.04 (0.74–1.45) | 1.25 (0.76–2.03) | 1.34 (1.08–1.67) |
| 39–40 | Ref. | Ref. | Ref. | Ref. | Ref. | Ref. | Ref. |
| CP, cerebral palsy. | | | | | | | |
| ^a^ Rate is calculated as number of cases per 10 000 person-years. | | | | | | | |
| ^b^ Model adjusted for maternal age at childbirth, parity, educational level, country of birth, smoking during pregnancy, height, early pregnancy BMI, diabetic diseases, hypertensive diseases, chorioamnionitis, infectious diseases during pregnancy, placental abruption, mode of delivery, and child's sex, major malformation, birth weight for gestational age, and calendar year of birth. | | | | | | | |

**Supplementary Table S6. Gestational age and neonatal morbidity (potential mediators) in children born at 22–40 weeks in Sweden during 1998–2016**

| **Neonatal morbidity (mediators)** | **Gestational age (weeks)** | | | | | | |  |
| --- | --- | --- | --- | --- | --- | --- | --- | --- |
|  | **22–24** | **25–27** | **28–31** | **32–34** | **35–36** | **37–38** | **39–40** | **All (22–40)** |
| **No. of children (%)^a^** | 832 (0.06) | 2744 (0.20) | 8063 (0.58) | 21 975 (1.57) | 56 011 (3.99) | 346 859 (24.74) | 965 756 (68.87) | 1 402 240 (100.00) |
| **Asphyxia** |  |  |  |  |  |  |  |  |
| **Intrapartum hypoxia** |  |  |  |  |  |  |  |  |
| No. of cases (%) | 1 (0.12) | 3 (0.11) | 21 (0.26) | 37 (0.17) | 48 (0.09) | 138 (0.04) | 418 (0.04) | 666 (0.05) |
| **Birth asphyxia** |  |  |  |  |  |  |  |  |
| No. of cases (%) | 203 (24.40) | 416 (15.16) | 662 (8.21) | 898 (4.09) | 1051 (1.88) | 2505 (0.72) | 7030 (0.73) | 12 765 (0.91) |
| **Any asphyxia** |  |  |  |  |  |  |  |  |
| No. of cases (%) | 204 (24.52) | 419 (15.27) | 676 (8.38) | 918 (4.18) | 1072 (1.91) | 2592 (0.75) | 7268 (0.75) | 13 149 (0.94) |
| **Respiratory-related diseases** |  |  |  |  |  |  |  |  |
| **Respiratory distress syndrome** |  |  |  |  |  |  |  |  |
| No. of cases (%) | 704 (84.62) | 2040 (74.34) | 3248 (40.28) | 2117 (9.63) | 807 (1.44) | 373 (0.11) | 236 (0.02) | 9525 (0.68) |
| **Bronchopulmonary dysplasia** |  |  |  |  |  |  |  |  |
| No. of cases (%) | 439 (52.76) | 1075 (39.18) | 645 (8.00) | 61 (0.28) | 11 (0.02) | 12 (0.00) | 28 (0.00) | 2271 (0.16) |
| **Any respiratory-related diseases** |  |  |  |  |  |  |  |  |
| No. of cases (%) | 757 (90.99) | 2224 (81.05) | 3416 (42.37) | 2137 (9.72) | 816 (1.46) | 383 (0.11) | 251 (0.03) | 9984 (0.71) |
| **Infection-/inflammatory-related diseases** |  |  |  |  |  |  |  |  |
| **Sepsis** |  |  |  |  |  |  |  |  |
| No. of cases (%) | 343 (41.23) | 830 (30.25) | 605 (7.50) | 295 (1.34) | 200 (0.36) | 422 (0.12) | 981 (0.10) | 3676 (0.26) |
| **Other infections specific to the perinatal period** |  |  |  |  |  |  |  |  |
| No. of cases (%) | 59 (7.09) | 185 (6.74) | 449 (5.57) | 759 (3.45) | 751 (1.34) | 1636 (0.47) | 4159 (0.43) | 7998 (0.57) |
| **Pneumonia** |  |  |  |  |  |  |  |  |
| No. of cases (%) | 44 (5.29) | 117 (4.26) | 129 (1.60) | 209 (0.95) | 294 (0.52) | 835 (0.24) | 2697 (0.28) | 4325 (0.31) |
| **Central nervous system inflammatory diseases** |  |  |  |  |  |  |  |  |
| No. of cases (%) | 5 (0.60) | 8 (0.29) | 15 (0.19) | 14 (0.06) | 14 (0.02) | 72 (0.02) | 157 (0.02) | 285 (0.02) |
| **Necrotizing enterocolitis** |  |  |  |  |  |  |  |  |
| No. of cases (%) | 90 (10.82) | 206 (7.51) | 130 (1.61) | 58 (0.26) | 18 (0.03) | 32 (0.01) | 32 (0.00) | 566 (0.04) |
| **Any infection-/inflammatory-related diseases** |  |  |  |  |  |  |  |  |
| No. of cases (%) | 445 (53.49) | 1135 (41.36) | 1221 (15.14) | 1271 (5.78) | 1229 (2.19) | 2909 (0.84) | 7831 (0.81) | 16 041 (1.14) |
| **Neurological-related diseases** |  |  |  |  |  |  |  |  |
| **Convulsion** |  |  |  |  |  |  |  |  |
| No. of cases (%) | 19 (2.28) | 43 (1.57) | 77 (0.95) | 117 (0.53) | 172 (0.31) | 585 (0.17) | 1447 (0.15) | 2460 (0.18) |
| **Intracranial nontraumatic haemorrhage** |  |  |  |  |  |  |  |  |
| No. of cases (%) | 317 (38.10) | 528 (19.24) | 625 (7.75) | 194 (0.88) | 93 (0.17) | 183 (0.05) | 345 (0.04) | 2285 (0.16) |
| **Periventricular leukomalacia** |  |  |  |  |  |  |  |  |
| No. of cases (%) | 21 (2.52) | 33 (1.20) | 64 (0.79) | 25 (0.11) | 18 (0.03) | 19 (0.01) | 17 (0.00) | 197 (0.01) |
| **Any neurological-related diseases** |  |  |  |  |  |  |  |  |
| No. of cases (%) | 327 (39.30) | 561 (20.44) | 702 (8.71) | 305 (1.39) | 247 (0.44) | 690 (0.20) | 1600 (0.17) | 4432 (0.32) |
| **Any of the above morbidity** |  |  |  |  |  |  |  |  |
| No. of cases (%) | 780 (93.75) | 2436 (88.78) | 4408 (54.67) | 4015 (18.27) | 3074 (5.49) | 6126 (1.77) | 15779 (1.63) | 36 618 (2.61) |

^a^ row percentage

| **Supplementary Table S7. Gestational age and risk of cerebral palsy before 5 years of age in children born at 22-40 weeks in Sweden during 1998-2016** | | | | | | | |
| --- | --- | --- | --- | --- | --- | --- | --- |
| **Gestational age (weeks)** | **No. of children (%)** | **Person years** | **Cerebral Palsy** | | | | |
|  |  |  | **No. of cases** | **Rate^a^** | **Hazard Ratio (95%CI)** | | |
|  |  |  |  |  | **Model 1^b^** | **Model 2^c^** | **Model 3^d^** |
| **As continuous variable (per one week decrease)** | 1 402 240 (100.00) | 6 186 862 | 2667 | 4.31 | 1.37 (1.36–1.38) | 1.37 (1.36–1.39) | 1.30 (1.28–1.32) |
| **As categorical variable** |  |  |  |  |  |  |  |
| 22–24 | 832 (0.06) | 2985 | 72 | 241.23 | 88.26 (69.25–112.49) | 83.42 (61.49–113.18) | 50.04 (35.42–70.71) |
| 25–27 | 2744 (0.20) | 11 216 | 176 | 156.92 | 60.60 (51.65–71.10) | 58.87 (49.03–70.69) | 26.62 (21.17–33.46) |
| 28–31 | 8063 (0.58) | 34 682 | 315 | 90.83 | 35.63 (31.42–40.41) | 33.63 (29.21–38.71) | 17.32 (14.33–20.92) |
| 32–34 | 21 975 (1.57) | 96 743 | 250 | 25.84 | 10.09 (8.79–11.59) | 9.11 (7.80–10.63) | 6.09 (5.13–7.23) |
| 35–36 | 56 011 (3.99) | 248 586 | 193 | 7.76 | 3.04 (2.60–3.54) | 2.92 (2.47–3.44) | 2.32 (1.96–2.75) |
| 37–38 | 346 859 (24.74) | 1 537 647 | 566 | 3.68 | 1.44 (1.30–1.59) | 1.42 (1.28–1.58) | 1.29 (1.15–1.44) |
| 39–40 | 965 756 (68.87) | 4 255 003 | 1095 | 2.57 | Ref. | Ref. | Ref. |
| ^a^ Rate is calculated as number of cases per 10 000 person-years. | | | | | | | |
| ^b^ Unadjusted model. | | | | | | | |
| ^c^ Model 2 adjusted for maternal age at childbirth, parity, educational level, country of birth, smoking during pregnancy, height, and early pregnancy BMI. | | | | | | | |
| ^d^ Model 3 adjusted for maternal age at childbirth, parity, educational level, country of birth, smoking during pregnancy, height, early pregnancy BMI, diabetic diseases, hypertensive diseases, chorioamnionitis, infectious diseases during pregnancy, placental abruption, mode of delivery, and child's sex, major malformation, birth weight for gestational age, and calendar year of birth. | | | | | | | |
